# Inhibition of Orbivirus Replication by Aurintricarboxylic Acid

**DOI:** 10.3390/ijms21197294

**Published:** 2020-10-02

**Authors:** Celia Alonso, Sergio Utrilla-Trigo, Eva Calvo-Pinilla, Luis Jiménez-Cabello, Javier Ortego, Aitor Nogales

**Affiliations:** Animal Health Research Centre (CISA), National Institute for Agriculture and Food Research and Technology (INIA), Valdeolmos, 28130 Madrid, Spain; alonsocelia01@gmail.com (C.A.); sergioutrilla@gmail.com (S.U.-T.); calvo.eva@inia.es (E.C.-P.); luisfjim@ucm.es (L.J.-C.)

**Keywords:** *Orbivirus*, Arbovirus, Bluetongue virus, African horse sickness virus, aurintricarboxylic acid, antiviral

## Abstract

Bluetongue virus (BTV) and African horse sickness virus (AHSV) are vector-borne viruses belonging to the *Orbivirus* genus, which are transmitted between hosts primarily by biting midges of the genus *Culicoides*. With recent BTV and AHSV outbreaks causing epidemics and important economy losses, there is a pressing need for efficacious drugs to treat and control the spread of these infections. The polyanionic aromatic compound aurintricarboxylic acid (ATA) has been shown to have a broad-spectrum antiviral activity. Here, we evaluated ATA as a potential antiviral compound against *Orbivirus* infections in both mammalian and insect cells. Notably, ATA was able to prevent the replication of BTV and AHSV in both cell types in a time- and concentration-dependent manner. In addition, we evaluated the effect of ATA in vivo using a mouse model of infection. ATA did not protect mice against a lethal challenge with BTV or AHSV, most probably due to the in vivo effect of ATA on immune system regulation. Overall, these results demonstrate that ATA has inhibitory activity against *Orbivirus* replication in vitro, but further in vivo analysis will be required before considering it as a potential therapy for future clinical evaluation.

## 1. Introduction

Viruses of the *Orbivirus* genus in the Reoviridae family are non-enveloped with an icosahedral capsid formed by three concentric protein layers, enclosing a double-stranded viral RNA genome formed by 10 segments [1,2]. The viral genome encodes seven structural proteins and six nonstructural proteins [1,2,3,4]. Orbiviruses can infect a wide host range, such as ruminants, equids, camelids, marsupials, sloths, seabirds, bats, snakes, and in some cases humans [5,6,7,8,9]. The two most relevant orbiviruses in animal health are Bluetongue virus (BTV) and African horse sickness virus (AHSV), which are arboviruses transmitted between their mammalian hosts mainly through *Culicoides* spp. that function as vectors. BTV is classified as a species of the genus *Orbivirus,* within the family Reoviridae, and 29 serotypes have been identified. BTV has a very wide distribution and can cause a severe hemorrhagic disease in ruminants, principally in sheep [10,11,12,13,14]. In addition, BTV can cause abortion or deformities in lambs or calves [15,16,17]. Multiple outbreaks of BTV all over the world have been documented, causing important economic losses [18,19,20,21]. On the other hand, AHSV causes acute disease in equids, where mortality can reach 90% in susceptible horses [22,23,24]. There are nine serotypes distributed mostly in Sub-saharan Africa, although cases have been reported in Asia and southern Europe [23,25,26].

Vaccination is considered the most effective approach to protect against BTV or AHSV infections [12,26,27,28,29,30,31]. However, the control of these viral infections is a difficult task due to uncontrolled vector propagation, the number of circulating serotypes in combination with the lack of cross-protection between them, and the wide host range [5,6,7,8,9,25,32]. Moreover, the impact of BTV and AHSV infections is increased by the risk of sporadic epidemics in regions where the viruses were not circulating previously. Although vaccination is an effective approach to prevent infections, this prophylactic method takes from two weeks to several months to show protection [33,34]. Therefore, vaccination is not the most suitable method for animals that have already been infected or to control a new outbreak. Importantly, antivirals for the treatment of infections caused by BTV or AHSV could help in the control of epidemics, reducing the spread of the disease as well as the economic impact typically associated with those outbreaks.

**Figure 1:**
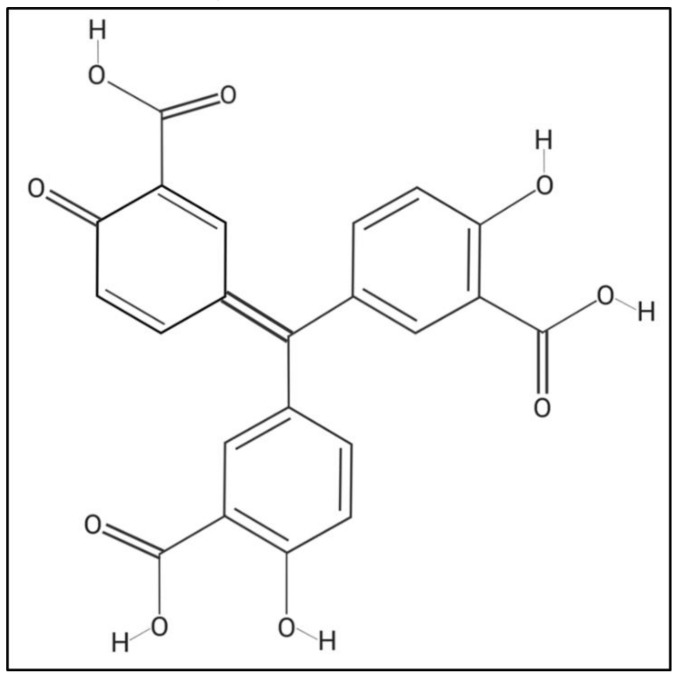
Chemical structure of aurintricarboxylic Acid (ATA). Molecular weight = 422.3 g/mol. Compound ID in PubChem 2259.

Aurintricarboxylic acid (ATA), which is a polyanionic aromatic compound with the chemical formula C_22_H_14_O_9_ (Figure 1) [35], has been shown to have multiple effects at the cellular level, such as being an antagonist of type 1 insulin growth factor receptor (IGF-1) [36,37], blocking the binding site of ribonuclease enzymes and topoisomerase II to nucleic acids [38,39], inhibiting the initiation of protein synthesis and apoptosis [40,41,42], among others [43,44]. Interestingly, it has been described that ATA, through different mechanisms of action, is able to inhibit the infection of multiple RNA and DNA viruses, including Human Immunodeficiency Virus (HIV) [45], Enterovirus 71 (EV71) [46], Severe Acute Respiratory Syndrome coronavirus (SARS-CoV) [47,48], influenza A and B viruses [49,50], Dengue virus (DENV) [51,52], Zika virus (ZIKV) [35], Hepatitis C virus (HCV) [53,54,55], and Vaccinia virus (VV) [56,57,58]. Because ATA possess a broad-spectrum antiviral activity, we have evaluated this compound as a therapeutic alternative against different members of the genus *Orbivirus*. Our data indicate that ATA efficiently inhibits the replication of BTV and AHSV in mammalian and insect cells, although it cannot prevent the infection when used as prophylactic measure. When we evaluated the antiviral activity of ATA in a mouse model of infection, the compound exacerbated the viral disease, most probably because of its toxicity, which could be associated with changes in immune system homeostasis. Overall, our results suggest that ATA could be a suitable alternative to control *Orbivirus* infections, but further studies should be conducted to understand and reduce its in vivo toxicity.

## 2. Results

### 2.1. Inhibitory Effect of ATA on Viral Replication

**Figure 2:**
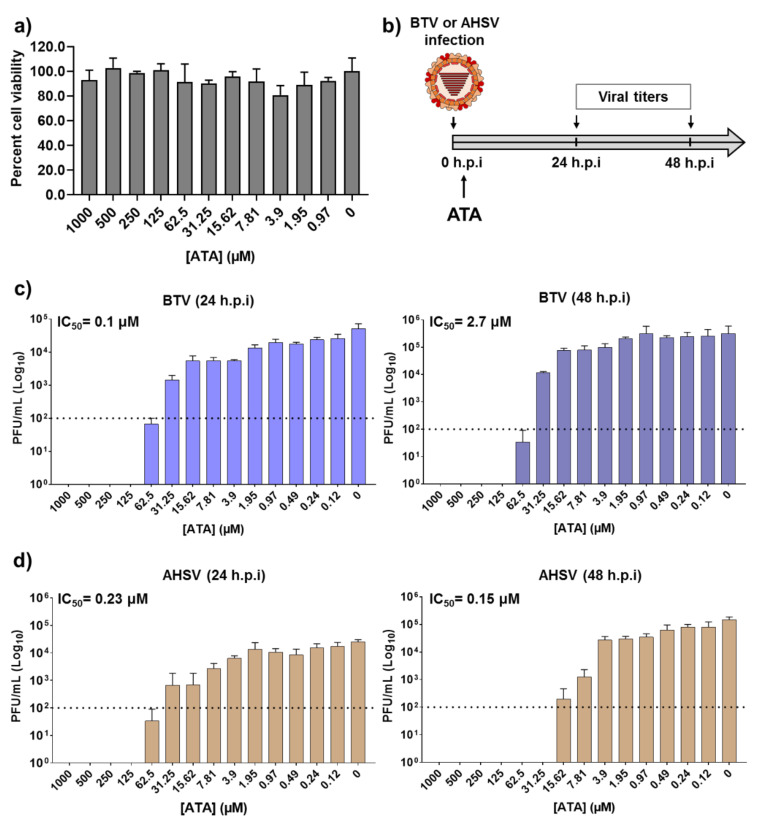
Inhibition of Bluetongue virus (BTV) and African horse sickness virus (AHSV) by ATA. (**a**) Cytotoxicity of ATA. Vero cells were treated with the indicated doses (two-fold dilutions, starting concentration 1000 μM) of ATA. Cell viability was analyzed at 24 h after treatment. Data are expressed as mean and SD. (**b**) Schematic representation of the experimental design. Vero cells were infected (multiplicity of infection (MOI), 0.01) with BTV-4 (**c**) or AHSV-4 (**d**), and after 90 min of viral adsorption, the indicated concentrations (two-folds dilutions, starting concentration 1000 μM) of ATA were added to the infection media (0 h.p.i.). At 24 (left) and 48 (right) h post-infection (h.p.i)., tissue culture supernatants were collected for virus titration by the plaque assay. The IC_50_ is indicated. Data are expressed as mean and SD. Dotted lines indicate the limit of detection.

Before examining the inhibitory effect of ATA (Figure 1) against BTV or AHSV infection, we first determined the toxicity of ATA in Vero cells (Figure 2a). To do this, we treated cells with serial (two-fold) dilutions of ATA and measured cell viability at 24 h post-treatment using the MTT colorimetric assay. As expected and based on previous reports [35], we did not observe any toxic effect of ATA, even at the highest concentration tested (1000 μM). We found that the 50% cytotoxic concentration (CC_50_) was >1000 μM.

To determine the therapeutic activity of ATA, Vero cells were infected with BTV-4 or AHSV-4 using a multiplicity of infection (MOI) of 0.01. After 90 min of viral absorption, the virus inoculum was replaced with infection medium containing two-fold serial dilutions (starting concentration of 1000 μM) of ATA. At 24 or 48 h post-infection (h.p.i), tissue culture supernatants were collected, viral titers were measured by the plaque assay, and the 50% inhibitory concentration (IC_50_) for each virus was calculated (Figure 2b). Viral replication was inhibited in a dose-dependent manner, and the calculated IC_50_ values for BTV-4 were 0.1 and 2.7 µM, at 24 or 48 hpi, respectively (Figure 2c). In addition, the IC_50_ for AHSV-4 at 24 and 48 h.p.i were 0.23 and 0.15 µM, respectively (Figure 2d). Importantly, the IC_50_ values obtained are similar to those reported for other viral systems [35,45,46,47,48,51,53,54,55,56,57,58]. These data indicate that ATA exhibited an effective and potent inhibition of BTV-4 and AHSV-4 infections, suggesting a potential use of ATA as a therapeutic method to treat animals infected by orbiviruses.

**Figure 3:**
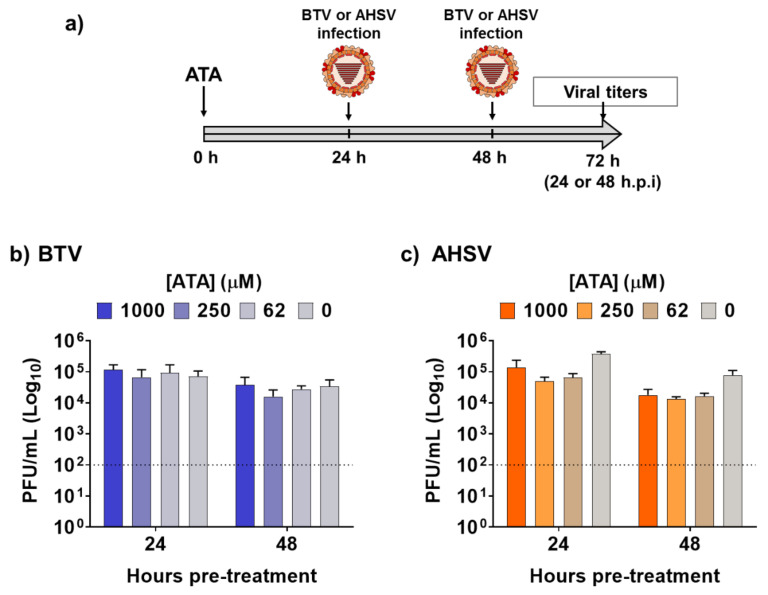
Pre-treatment with ATA did not prevent infection by BTV or AHSV. (**a**) Schematic representation of the experimental design. Vero cells were pre-treated with the indicated concentrations of ATA for 24 or 48 h before infection (MOI, 0.01) with BTV (**b**) or AHSV (**c**). At 24 h.p.i, tissue culture supernatants were collected for virus titration by the plaque assay. Data re expressed as mean and SD. Dotted lines indicate the limit of detection.

Next, we assessed the feasibility of using ATA for the prevention of infection caused by BTV-4 or AHSV-4 (Figure 3a), which could be important for the rapid control of potential epidemics, or as a preventive measure when animals are moved to regions where these viruses are endemic. To that end, Vero cells were pre-treated with different concentrations of ATA for 24 or 48 h prior to infection (MOI, 0.01) with BTV-4 (Figure 3b) or ASHV-4 (Figure 3c). At 24 h.p.i, viral replication from tissue culture supernatants was evaluated by plaque assay. No significant differences in BTV-4 or AHSV-4 viral titers were detected in ATA-treated cells compared to control cells, indicating that ATA was not able to prevent infection by BTV-4 or AHSV-4 and, thus, that ATA is not an effective prophylactic alternative chemical against orbiviruses.

### 2.2. ATA Testing in Mice

**Figure 4:**
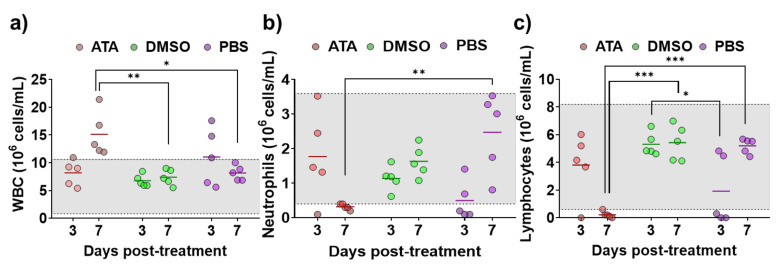
Toxicity of ATA in uninfected mice. Evaluation of blood parameters in samples taken from IFNAR ^(−/−)^ mice treated with ATA (red), DMSO (green), and PBS (purple) to determine the number of white blood cells (WBC) (**a**), neutrophils (Neu) (**b**), and lymphocytes (Lym) (**c**). Samples were evaluated with a multiparameter autohematology analyzer at 3 and 7 days post-treatment. * *p* < 0.05, ** *p* < 0.005, *** *p* < 0.0005, using two-way ANOVA. Horizontal lines indicate the means. Gray shadows indicates reference values for selected hematological parameters.

Even if it has been shown that ATA has in vitro inhibitory activity against multiple RNA or DNA viruses [35,45,46,47,48,51,53,54,55,56,57,58], its antiviral efficacy in in vivo models has not been demonstrated [58], and there is a limited number of studies showing the effect of ATA in vivo [41,59,60]. Therefore, prior to conducting antiviral experiments in IFNAR ^(−/−)^ mice, a well-accepted mouse model to study BTV and AHSV infections [61,62], the effect of ATA in this animal model was assessed. For that, three groups of animals were treated daily for eight days with 600 µg of ATA diluted in PBS, DMSO diluted in PBS, or PBS. The indicated solutions were injected intraperitoneally (i.p.), and mice were daily evaluated for clinical signs of toxicity and mortality. All animals survived the different treatments and did not display behaviors or clinical signs that could suggest drug toxicity. In addition, at 3 and 7 days post-treatment, blood samples were collected, and a hematological analysis was performed (Figure 4). A significant increase in white blood cells (Figure 4a) and a significant reduction in neutrophils (Figure 4b) and lymphocytes (Figure 4c) were observed at 7 days post-treatment in mice treated with ATA. Thus, data suggest that ATA could have an effect on the physiology and immune response of animals as it causes leukocytosis, neutrophilia, and lymphopenia, even if the mice do not show clinical signs of toxicity.

**Figure 5:**
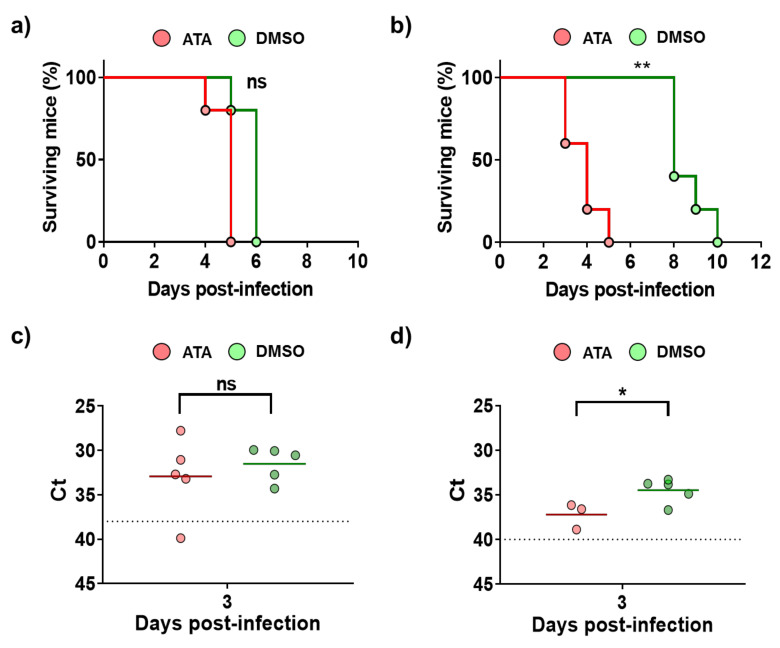
Analysis of ATA activity in mice. Groups of IFNAR ^(−/−)^ mice were treated with ATA or DMSO and challenged with a lethal dose of BTV-4 (**a**,**c**) or AHSV-4 (**b**,**d**). Treatments began 24 h before virus exposure and were administered i.p once daily for 8 days or until the mice died. (**a**,**b**) Effect of ATA on survival. Mortality of ATA- or DMSO-treated mice challenged with BTV-4 (**a**) or AHSV-4 (**b**) was evaluated. Log rank (Mantel–Cox) test was used to compare groups. ** *p* < 0.005 ns, not significant. (**c**,**d**) Effect of ATA on viral replication. Blood samples were collected on day 3 post-infection for subsequent analysis of viremia by real-time RT-qPCR. Horizontal lines indicate the means. Dotted lines indicate the threshold of a Ct of 38 for BTV-4 or 40 for AHSV-4, above which viremia is considered absent. * *p* < 0.05, using Student’s *t*-test.

Next, antiviral studies were conducted in mice infected with BTV-4 or AHSV-4 (Figure 5). To that end, IFNAR ^(−/−)^ mice were divided in four groups: groups 1 and 3 were treated with 600 µg of ATA dissolved in PBS, and groups 2 and 4 (controls) were administered DMSO dissolved in PBS. Inoculations were carried out for eight days or until the mice died, starting one day before infection. Groups 1 and 2 were subcutaneously infected with BTV-4 (10 PFU/mouse), and groups 3 and 4 were subcutaneously infected with AHSV-4 (5 × 10^5^ PFU/mouse). Mortality and the appearance of clinical signs were evaluated daily. The treatment with ATA did not prevent death of the animals infected with BTV-4 (Figure 5a), and usually animals that received the drug died earlier, although differences were not significant. This pattern was most evident for AHSV-4, as mice in the control group succumbed to infection about five days later than animals treated with ATA (Figure 5b). The observation of clinical signs of infection correlated with the mortality rates. To evaluate viral replication, the presence of the virus in the blood was measured by RT-qPCR at 3 d.p.i (Figure 5c,d). Contrary to what was expected based on the observed mortality rates, in general, animals in control groups, which were not treated with ATA, showed higher levels of viremia for AHSV-4 (Figure 5d). However, differences were not observed between groups of mice inoculated with BTV (Figure 5c). These results suggest that ATA could be able to inhibit viral infection in IFNAR ^(−/−)^ mice. However, due to the observed toxic effect of ATA in vivo (Figure 4), which jeopardizes the physiology and immune response of animals, this compound is not a therapeutic option, at least in the current formulation.

### 2.3. Therapeutic Activity of ATA in Insect Cells

**Figure 6:**
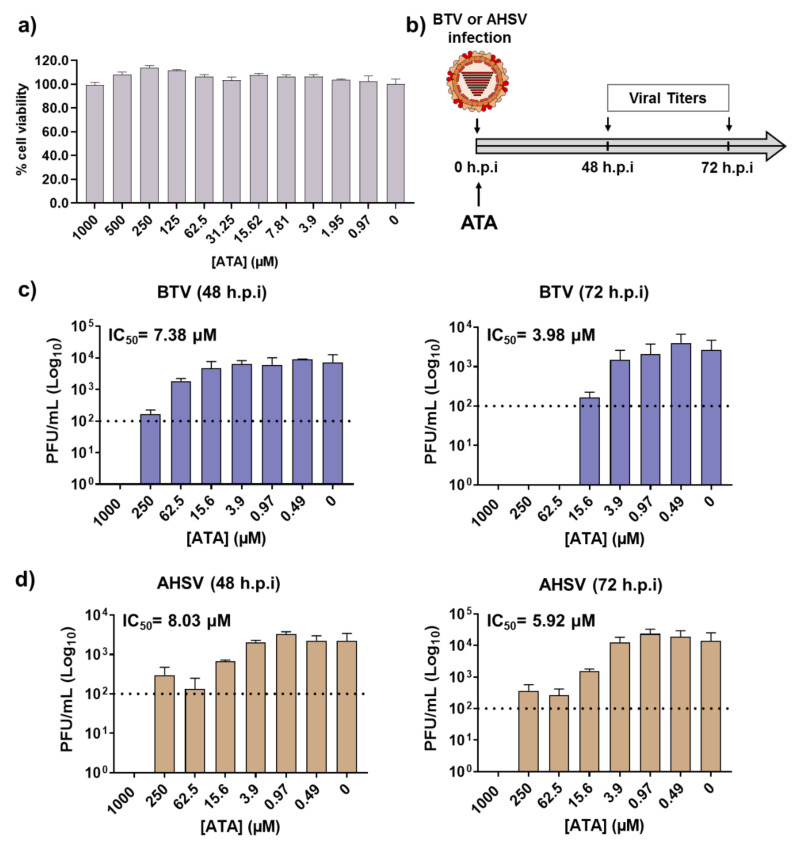
Antiviral activity of ATA in insect KC cells. (**a**) Cytotoxicity of ATA. KC cells were treated with the indicated doses (two-fold dilutions, starting concentration 1000 μM) of ATA, and cell viability was analyzed. Data are expressed as mean and SD. (**b**) Schematic representation of the experimental design. Cells were infected with BTV-4 (**c**) or AHSV-4 (**d**) at MOI of 0.01 and treated with the indicated concentrations of ATA. At 48 and 72 h.p.i, viral replication was evaluated by the plaque assay. Dotted lines indicate the limit of detection.

BTV and AHSV are arboviruses transmitted between their animal hosts via the bites of hematophagous midges of the genus *Culicoides*. Like all arboviruses, these viruses have the ability to infect and propagate in multiple cell types, which allows them to productively infect both mammalian and insect hosts. Given that ATA was able to inhibit BTV-4 and AHSV-4 replication in mammalian cells, we evaluated whether the drug could inhibit viral growth in insect cells, which may be important to control the spread of BTV and AHSV through their insect vectors. Even at the highest concentration tested of ATA (1000 μM), we did not detect any toxicity in KC insect cells (Figure 6a). Therefore, KC cells were infected with BTV-4 or AHSV-4 (MOI, 0.01), and after 90 min of viral absorption, the viral inoculum was replaced by fresh medium containing different concentrations of ATA. Samples of the supernatants were collected at 48 and 72 h.p.i to assess viral replication by the plaque assay (Figure 6b). The compound showed antiviral activity against BTV-4 (Figure 6c) and AHSV-4 (Figure 6d) at different times, demonstrating a dose-dependent inhibition of viral replication. The IC_50_ calculated for BTV-4 at 48 and 72 h.p.i were 7.38 and 3.98 µM, respectively. On the other hand, the determined IC_50_ for AHSV-4 at 48 and 72 h.p.i were 8.03 and 5.92 µM, respectively. These data indicate that ATA exhibits an effective inhibition of BTV and AHSV infection in insect cells.

## 3. Discussion

During the last decades, various members of the *Orbivirus* genus, including BTV and AHSV, have expanded their geographical distribution, causing numerous infectious outbreaks in new regions where they were not endemic. Orbiviruses are transmitted by arthropod vectors, an added difficulty for their control, as climate change together with globalization contribute to the expansion of these vectors and to their establishment in new areas [25,63,64]. Orbiviruses, such as BTV and AHSV, cause serious damage to animal health and have a significant economic impact on society [9,23,26]. Currently, the best approach to prevent BTV and AHSV infections is vaccination, although this approach presents multiple limitations, and vaccine strategies need to be improved [12,26,27,28,29,30,31]. More importantly, although vaccines can prevent infections creating protective immunity, they are not an effective method to treat infected animals, which usually need to be sacrificed causing important economic losses. Therefore, there is an urgent need to develop new therapeutic strategies, including the identification of antivirals, for the control of infections by these viral pathogens.

There are no specific antiviral drugs against BTV or AHSV. Li et al., used a cytopathic effect (CPE)-based high-throughput screening (HTS) assay to identified compounds with antiviral activity against BTV-10 infection in BSR cells [65]. The authors identified compounds with IC_50_ lower than 100 μM, including several mononuclear heterocyclic compounds such as thiophene and thiazole derivatives or bicyclic compounds such as pyrimidines fused to a 1,3,4-thiadiazole, 1,2-4-thiadiazole, and pyrazole ring systems [65]. Recently, Sekhar et al., synthesized new series of phosphorylated abacavir derivatives, which were tested for antiviral activity against BTV using embryonated eggs and BHK-21 cells [66]. The authors identified some drugs with antiviral activity against BTV when used at high concentrations, and the IC_50_ were not determined [66]. Notably, in any of these reports, the authors evaluated the cytotoxicity or the in vivo activity of the identified compounds [65,66].

ATA has shown a potent antiviral activity against numerous RNA or DNA viruses [35,45,46,47,48,51,53,54,55,56,57,58]. Thus, we tested the hypothesis that ATA could also be a potent antiviral candidate against orbiviruses such as BTV and AHSV. We demonstrated that ATA has an effective therapeutic and dose-dependent antiviral activity against BTV-4 and AHSV-4 replication (Figure 2) in mammalian Vero cells, showing similar IC_50_ to those obtained for other viruses [35,45,46,47,48,51,53,54,55,56,57,58]. However, our results indicated that pre-treatment with ATA does not inhibit infection by BTV-4 and AHSV-4 (Figure 3). The saliva of *Culicoides* may play a role in viral replication and transmission by recruiting leucocytes where the virus can replicate or by generating infectious subviral particles, thus enhancing particle infectivity [63,64,67]. In this study, we demonstrated that ATA is able to inhibit viral replication in insect cells (Figure 6), which could be important to prevent and control the spread of these viruses through their insect vectors. Although ATA has been reported to affect the function of multiple viral or host proteins [39,40,47,49,55,57], the exact mechanism by which ATA inhibits BTV-4 or AHSV-4 replication was not identified, and further studies will be required to determine if ATA is able to interact with viral proteins and block specific steps during viral infection.

ATA has been amply evaluated and characterized in tissue culture cells, but limited findings have been reported about its potential antiviral activity in vivo [58]. In the case of Vaccinia virus, the tolerated dose of ATA (~10 or 30 mg/kg) failed to protect BALB/c mice from a lethal challenge. Indeed, this drug accelerated the time to death compared with a placebo, indicating that ATA exacerbated the infection [58]. Similarly, in our study, ATA was not able to protect IFNAR ^(−/−)^ mice against a lethal challenge with BTV-4 or AHSV-4, and the drug decreased the survival time of infected mice compared with untreated infected mice (Figure 5). Interestingly, a significant reduced viremia was observed in animals treated with ATA and infected with AHSV-4 (Figure 5), suggesting inhibition of viral replication mediated by ATA. Notably, differences observed for the effect of ATA on the infection for BTV or AHSV (in vitro and in vivo) could help to determine the mechanism of action of ATA. In addition, the effect of ATA in IFNAR ^(−/−)^ mice was examined, showing changes in several blood parameters. Leukocytosis, neutropenia, and lymphopenia were observed in ATA-treated animals (Figure 4), which could have important consequences to fight the viral infection [11,68,69], providing an explanation for the lack of increased survival rates upon ATA administration. In fact, ATA has been reported to block the JAK/STAT signaling pathway, which is critical for Th cell differentiation [41]. In this study, the authors indicated that ATA treatment blocked chemotaxis and accumulation of dendritic cells in the spleen of mice. In addition, ATA was found to inhibit the functions of many chemokine receptors, which suggests it may be effective in treating autoimmune diseases [41]. However, additional studies are necessary to evaluate the toxicity of ATA and its antiviral activity in vivo. Additionally, new analyses using other animal models, including natural hosts, or ATA-like compounds would be highly desired.

## 4. Materials and Methods

### 4.1. Cells and Viruses

African green monkey kidney epithelial Vero cells (ATCC, Cat No. CCL-81) were grown in Dulbecco’s modified Eagle’s medium (DMEM) supplemented with 50 mg/mL gentamicin, 10 mg/mL penicillin/streptomycin, 1% non-essential amino acids, 2 mM l-glutamine, and 10% heat-inactivated fetal bovine serum (FBS) at 37 °C in a 5% CO_2_ atmosphere. KC insect cells, obtained from *Culicoides. sonorensis* larvae, were grown in Schneider’s insect medium supplemented as indicated above, at 29 °C. BTV serotype 4 (MOR2009/09) (BTV-4) was isolated from sheep’s blood and raised in KC insect cells [13,61]. A Spanish isolate of AHSV serotype 4 (Madrid-87) was passaged twice in mouse brain and three times in BHK-21 [70]. Virus stocks were propagated in Vero cells and titrated by the plaque assay as previously described [27,28,71]. KC cells and BTV-4(M) were generously provided by Professor Peter Mertens (IAH-Pirbright, UK).

### 4.2. Compounds

ATA was acquired from Sigma-Aldrich (Sigma Aldrich, St. Louis, MO, USA. Catalog No. A1895). The compound was prepared as a 100 mM stock solution in dimethyl sulfoxide (DMSO) [35] and kept at −20 °C until experimental use.

### 4.3. Cell Viability Assay

Viability of mammalian Vero and insect KC cells was measured using the MTT Cell Proliferation and Cytotoxicity Assay Kit (Boster Biological Technology, Pleasanton, CA 9, USA) following the manufacturer’s instructions. Briefly, confluent cells (96-well plate format, 5 × 10^4^ cells/well, triplicates) were treated with 100 μL of medium containing the indicated concentrations of ATA (two-fold dilutions, starting concentration of 1000 μM). Plates were incubated at 37 °C or 29 °C for 24 h. Samples were treated with 15 μL of Dye Solution and incubated at 37 °C in a 5% CO_2_ atmosphere for 5 h. Next, the cells were treated with 100 μL of Formazan solubilisation solution for 16 h, and absorbance at 570 nm was read using a microplate reader. The viability of compound-treated cells was calculated as a percentage relative to values obtained with mock-treated cells. Non-linear regression curves and the median cytotoxic concentration (CC_50_) were calculated using GraphPad Prism software v. 8.0. (GraphPad Software, San Diego, CA, USA).

### 4.4. Antiviral Assays

#### 4.4.1. Therapeutic Activity

ATA-mediated inhibition of BTV or AHSV was evaluated in monolayers of Vero or KC cells (24-well plate format, 2 × 10^5^ cells/well, triplicates), which were infected with an MOI of 0.01. After 90 min of viral adsorption, the medium was replaced with 1 mL of infectious medium (containing only 2% FBS) supplemented with the indicated concentrations (starting concentration, 1000 μM) of ATA, and the cells were incubated at 37 °C (Vero cells) or 29 °C (KC cells). At 24 and 48 h.p.i. (Vero cells) or 48 and 72 h h.p.i (KC cells), aliquots of the tissue culture supernatants were collected and evaluated by the plaque assay as previously described [27,28,71]. Triplicate wells were used to calculate the mean and SD of the inhibition. The IC_50_ was determined by use of a sigmoidal dose–response curve (GraphPad Prism software v. 8.0).

#### 4.4.2. Prophylactic Activity

Vero cells (24-well plate format, 2 × 10^5^ cells/well, triplicates) were incubated with the indicated concentrations of ATA during 24 or 48 h. Then, the cells were infected (MOI, 0.01) with BTV or AHSV, and after 90 min of incubation at 37 °C, the medium was removed, and 1 mL of fresh infectious medium was added to the cells. At 24 h, tissue culture supernatants were harvested and evaluated by the plaque assay [27,28,71].

### 4.5. Mouse Experiments

Type I interferon receptor-defective mice (IFNAR ^(−/−)^) on a 129 Sv/Ev background were used for the experiments. Mice were housed under pathogen-free conditions in biosafety-level 3 (BSL3) animal facilities at the Animal Health Research Center (INIA-CISA), Madrid (Spain), before use. Male and female mice were used for the experiment, since previous studies have proved that the two genders do not show significant differences after infection by BTV-4 or AHSV-4 [72]. Animal experimental protocols were approved by the Ethical Review Committee at the INIA-CISA and Comunidad de Madrid (Permit number: PROEX 037/15), in strict accordance with EU guidelines 2010/63/UE about the protection of animals used for experimentation and the Spanish Animal Welfare Act 32/2007. Animals that showed severe clinical signs such as rough hair, reduced activity, eye swelling, or hypothermia were euthanized. Blood samples from the submandibular plexus were collected at the indicated days.

### 4.6. Analysis of the Effect of ATA In Vivo

Mice were divided in three groups (*n* = 15, 5 mice/group), and animals were inoculated i.p. with 200 µL of a solution containing: group 1, 600 µg of ATA (~30 mg/kg) diluted in PBS ([DMSO] 7.1%); group 2, DMSO diluted in PBS ([DMSO] 7.1%); group 3, PBS. Solutions were administrated once daily, during 8 days, and clinical signs of toxicity were monitored. After 3 and 7 days, blood samples were collected into EDTA tubes and evaluated using a multiparameter autohematology analyzer (BC-5300 Vet; Mindray, Shenzhen, China) [71].

### 4.7. Study of the Efficacy of ATA In Vivo

Mice were divided in four groups (*n* = 20, 5 mice/group). ATA or DMSO were administered once daily by the i.p. route, and treatment was commenced 1 day before infection and continued until death or day 8. Group 1 and 3 were treated with 600 µg of ATA (~30 mg/kg) diluted in PBS ([DMSO] 7.1%). Group 2 and 4 were used as controls and received DMSO diluted in PBS ([DMSO] 7.1%). One day after the first inoculation, groups 1 and 2 were challenged with 10 plaque-forming units (PFUs) of BTV, and groups 3 and 4 with 5 × 10^5^ PFUs of AHSV. Mice were subcutaneously inoculated with the indicated viruses diluted in PBS. After infection, mortality and clinical signs were evaluated daily. To determine viral replication, whole blood samples in EDTA were collected at 3 d.p.i from the challenged mice, and RNA was extracted using TRIzol (Invitrogen) following the manufacturer’s instructions. Viral titers were determined by real-time RT-qPCR specific for BTV segment 5 or AHSV segment 7, as described by Toussaint et al. [73] and Agüero et al. [74], respectively.

### 4.8. Statistical Analysis

Statistical analyses were carried out using GraphPad PRISM v. 8 (GraphPad Software, San Diego, CA, USA).

## References

[1] Roy P. (2017). Bluetongue virus structure and assembly. Curr. Opin. Virol..

[2] Patel A., Roy P. (2014). The molecular biology of Bluetongue virus replication. Virus Res..

[3] Stewart M., Hardy A., Barry G., Pinto R.M., Caporale M., Melzi E., Hughes J., Taggart A., Janowicz A., Varela M. (2015). Characterization of a second open reading frame in genome segment 10 of bluetongue virus. J. Gen. Virol..

[4] Drolet B.S., van Rijn P., Howerth E.W., Beer M., Mertens P.P. (2015). A Review of Knowledge Gaps and Tools for Orbivirus Research. Vector Borne Zoonotic Dis..

[5] Belaganahalli M.N., Maan S., Maan N.S., Nomikou K., Pritchard I., Lunt R., Kirkland P.D., Attoui H., Brownlie J., Mertens P.P.C. (2012). Full genome sequencing and genetic characterization of Eubenangee viruses identify Pata virus as a distinct species within the genus Orbivirus. PLoS ONE.

[6] Fagre A.C., Lee J.S., Kityo R.M., Bergren N.A., Mossel E.C., Nakayiki T., Nalikka B., Nyakarahuka L., Gilbert A.T., Peterhans J.K. (2019). Discovery and Characterization of Bukakata orbivirus (Reoviridae:Orbivirus), a Novel Virus from a Ugandan Bat. Viruses.

[7] Tomazatos A., Marschang R.E., Maranda I., Baum H., Bialonski A., Spînu M., Lühken R., Schmidt-Chanasit J., Cadar D. (2020). Letea Virus: Comparative Genomics and Phylogenetic Analysis of a Novel Reassortant Orbivirus Discovered in Grass Snakes (Natrix natrix). Viruses.

[8] Coetzee P., van Vuuren M., Venter E.H., Stokstad M. (2014). A review of experimental infections with bluetongue virus in the mammalian host. Virus Res..

[9] Maclachlan N.J., Guthrie A.J. (2010). Re-emergence of bluetongue, African horse sickness, and other orbivirus diseases. Vet. Res..

[10] Batten C.A., Henstock M.R., Steedman H.M., Waddington S., Edwards L., Oura C.A.L. (2013). Bluetongue virus serotype 26: Infection kinetics, pathogenesis and possible contact transmission in goats. Vet. Microbiol..

[11] Bréard E., Schulz C., Sailleau C., Bernelin-Cottet C., Viarouge C., Vitour D., Guillaume B., Caignard G., Gorlier A., Attoui H. (2018). Bluetongue virus serotype 27: Experimental infection of goats, sheep and cattle with three BTV-27 variants reveal atypical characteristics and likely direct contact transmission BTV-27 between goats. Transbound. Emerg. Dis..

[12] Calvo-Pinilla E., Castillo-Olivares J., Jabbar T., Ortego J., de la Poza F., Marín-López A. (2014). Recombinant vaccines against bluetongue virus. Virus Res..

[13] Nomikou K., Hughes J., Wash R., Kellam P., Breard E., Zientara S., Palmarini M., Biek R., Mertens P. (2015). Widespread Reassortment Shapes the Evolution and Epidemiology of Bluetongue Virus following European Invasion. PLoS Pathog..

[14] Zientara S., Sánchez-Vizcaíno J.M. (2013). Control of bluetongue in Europe. Vet. Microbiol..

[15] Wouda W., Peperkamp N.H.M.T., Roumen M.P.H.M., Muskens J., van Rijn A., Vellema P. (2009). Epizootic congenital hydranencephaly and abortion in cattle due to bluetongue virus serotype 8 in the Netherlands. Tijdschr. Diergeneeskd..

[16] Vinomack C., Rivière J., Bréard E., Viarouge C., Postic L., Zientara S., Vitour D., Belbis G., Spony V., Pagneux C. (2020). Clinical cases of Bluetongue serotype 8 in calves in France in the 2018-2019 winter. Transbound. Emerg. Dis..

[17] Chauhan H.C., Biswas S.K., Chand K., Rehman W., Das B., Dadawala A.I., Chandel B.S., Kher H.N., Mondal B. (2014). Isolation of bluetongue virus serotype 1 from aborted goat fetuses. Rev. Off. Int. Epizoot..

[18] Kundlacz C., Caignard G., Sailleau C., Viarouge C., Postic L., Vitour D., Zientara S., Breard E. (2019). Bluetongue Virus in France: An Illustration of the European and Mediterranean Context since the 2000s. Viruses.

[19] Gómez-Guillamón F., Caballero-Gómez J., Agüero M., Camacho-Sillero L., Risalde M.A., Zorrilla I., Villalba R., Rivero-Juárez A., García-Bocanegra I. (2020). Re-emergence of bluetongue virus serotype 4 in Iberian ibex (Capra pyrenaica) and sympatric livestock in Spain, 2018-2019. Transbound. Emerg. Dis..

[20] Rajko-Nenow P., Christodoulou V., Thurston W., Ropiak H.M., Savva S., Brown H., Qureshi M., Alvanitopoulos K., Gubbins S., Flannery J. (2020). Origin of Bluetongue Virus Serotype 8 Outbreak in Cyprus, September 2016. Viruses.

[21] Wilson A.J., Mellor P.S. (2009). Bluetongue in Europe: Past, present and future. Philos. Trans. R. Soc. Lond. B Biol. Sci..

[22] Lulla V., Losada A., Lecollinet S., Kerviel A., Lilin T., Sailleau C., Beck C., Zientara S., Roy P. (2017). Protective efficacy of multivalent replication-abortive vaccine strains in horses against African horse sickness virus challenge. Vaccine.

[23] Carpenter S., Mellor P.S., Fall A.G., Garros C., Venter G.J. (2017). African Horse Sickness Virus: History, Transmission, and Current Status. Annu. Rev. Entomol..

[24] Becker E., Venter G.J., Greyling T., Molini U., van Hamburg H. (2018). Evidence of African horse sickness virus infection of Equus zebra hartmannae in the south-western Khomas Region, Namibia. Transbound. Emerg. Dis..

[25] Mellor P.S., Boned J., Hamblin C., Graham S. (1990). Isolations of African horse sickness virus from vector insects made during the 1988 epizootic in Spain. Epidemiol. Infect..

[26] Dennis S.J., Meyers A.E., Hitzeroth I.I., Rybicki E.P. (2019). African Horse Sickness: A Review of Current Understanding and Vaccine Development. Viruses.

[27] Marín-López A., Otero-Romero I., de la Poza F., Menaya-Vargas R., Calvo-Pinilla E., Benavente J., Martínez-Costas J.M., Ortego J. (2014). VP2, VP7, and NS1 proteins of bluetongue virus targeted in avian reovirus muNS-Mi microspheres elicit a protective immune response in IFNAR(−/−) mice. Antivir. Res..

[28] Calvo-Pinilla E., Rodríguez-Calvo T., Sevilla N., Ortego J. (2009). Heterologous prime boost vaccination with DNA and recombinant modified vaccinia virus Ankara protects IFNAR(−/−) mice against lethal bluetongue infection. Vaccine.

[29] De la Poza F., Marín-López A., Castillo-Olivares J., Calvo-Pinilla E., Ortego J. (2015). Identification of CD8 T cell epitopes in VP2 and NS1 proteins of African horse sickness virus in IFNAR(−/−) mice. Virus Res..

[30] Calvo-Pinilla E., Marín-López A., Utrilla-Trigo S., Jiménez-Cabello L., Ortego J. (2020). Reverse genetics approaches: A novel strategy for African horse sickness virus vaccine design. Curr. Opin. Virol..

[31] Utrilla-Trigo S., Jiménez-Cabello L., Alonso-Ravelo R., Calvo-Pinilla E., Marín-López A., Moreno S., Lorenzo G., Benavides J., Gilbert S., Nogales A. (2020). Heterologous Combination of ChAdOx1 and MVA Vectors Expressing Protein NS1 as Vaccination Strategy to Induce Durable and Cross-Protective CD8+ T Cell Immunity to Bluetongue Virus. Vaccines.

[32] Federici V., Goffredo M., Mancini G., Quaglia M., Santilli A., Di Nicola F., De Ascentis M., Cabras P., Volpicelli C., De Liberato C. (2019). Vector Competence of Italian Populations of Culicoides for Some Bluetongue Virus Strains Responsible for Recent Northern African and European Outbreaks. Viruses.

[33] Anderson J., Hägglund S., Bréard E., Riou M., Zohari S., Comtet L., Olofson A.-S., Gélineau R., Martin G., Elvander M. (2014). Strong protection induced by an experimental DIVA subunit vaccine against bluetongue virus serotype 8 in cattle. Vaccine.

[34] Matsuo E., Celma C.C.P., Boyce M., Viarouge C., Sailleau C., Dubois E., Bréard E., Thiéry R., Zientara S., Roy P. (2011). Generation of replication-defective virus-based vaccines that confer full protection in sheep against virulent bluetongue virus challenge. J. Virol..

[35] Park J.-G., Ávila-Pérez G., Madere F., Hilimire T.A., Nogales A., Almazán F., Martínez-Sobrido L. (2019). Potent Inhibition of Zika Virus Replication by Aurintricarboxylic Acid. Front. Microbiol..

[36] Beery R., Haimsohn M., Wertheim N., Hemi R., Nir U., Karasik A., Kanety H., Geier A. (2001). Activation of the insulin-like growth factor 1 signaling pathway by the antiapoptotic agents aurintricarboxylic acid and evans blue. Endocrinology.

[37] Haimsohn M., Beery R., Karasik A., Kanety H., Geier A. (2002). Aurintricarboxylic acid induces a distinct activation of the IGF-I receptor signaling within MDA-231 cells. Endocrinology.

[38] Benchokroun Y., Couprie J., Larsen A.K. (1995). Aurintricarboxylic acid, a putative inhibitor of apoptosis, is a potent inhibitor of DNA topoisomerase II in vitro and in Chinese hamster fibrosarcoma cells. Biochem. Pharmacol..

[39] Catchpoole D.R., Stewart B.W. (1994). Inhibition of topoisomerase II by aurintricarboxylic acid: Implications for mechanisms of apoptosis. Anticancer Res..

[40] Roos A., Dhruv H.D., Mathews I.T., Inge L.J., Tuncali S., Hartman L.K., Chow D., Millard N., Yin H.H., Kloss J. (2017). Identification of aurintricarboxylic acid as a selective inhibitor of the TWEAK-Fn14 signaling pathway in glioblastoma cells. Oncotarget.

[41] Zhang F., Wei W., Chai H., Xie X. (2013). Aurintricarboxylic acid ameliorates experimental autoimmune encephalomyelitis by blocking chemokine-mediated pathogenic cell migration and infiltration. J. Immunol..

[42] Lim D.-G., Park Y.-H., Kim S.-E., Kim Y.-H., Park C.-S., Kim S.-C., Park C.-G., Han D.-J. (2011). Aurintricarboxylic acid promotes the conversion of naive CD4+CD25- T cells into Foxp3-expressing regulatory T cells. Int. Immunol..

[43] Lu H., Wei G., Wang D., Yung P., Ying W. (2008). Posttreatment with the Ca(2+)-Mg(2+)-dependent endonuclease inhibitor aurintricarboxylic acid abolishes genotoxic agent-induced nuclear condensation and DNA fragmentation and decreases death of astrocytes. J. Neurosci. Res..

[44] Hallock S., Tang S.-C., Buja L.M., Trump B.F., Liepins A., Weerasinghe P. (2007). Aurintricarboxylic acid inhibits protein synthesis independent, sanguinarine-induced apoptosis and oncosis. Toxicol. Pathol..

[45] Fong K., Smith T.J. (2009). Citrate-mediated release of aurintricarboxylic acid from a calcium alginate complex: Implications for intravaginal HIV chemoprophylaxis and related applications. Pharm. Dev. Technol..

[46] Hung H.-C., Chen T.-C., Fang M.-Y., Yen K.-J., Shih S.-R., Hsu J.T.-A., Tseng C.-P. (2010). Inhibition of enterovirus 71 replication and the viral 3D polymerase by aurintricarboxylic acid. J. Antimicrob. Chemother..

[47] Yap Y., Zhang X., Andonov A., He R. (2005). Structural analysis of inhibition mechanisms of aurintricarboxylic acid on SARS-CoV polymerase and other proteins. Comput. Biol. Chem..

[48] He R., Adonov A., Traykova-Adonova M., Cao J., Cutts T., Grudesky E., Deschambaul Y., Berry J., Drebot M., Li X. (2004). Potent and selective inhibition of SARS coronavirus replication by aurintricarboxylic acid. Biochem. Biophys. Res. Commun..

[49] Hashem A.M., Flaman A.S., Farnsworth A., Brown E.G., Van Domselaar G., He R., Li X. (2009). Aurintricarboxylic acid is a potent inhibitor of influenza A and B virus neuraminidases. PLoS ONE.

[50] Hung H.-C., Tseng C.-P., Yang J.-M., Ju Y.-W., Tseng S.-N., Chen Y.-F., Chao Y.-S., Hsieh H.-P., Shih S.-R., Hsu J.T.-A. (2009). Aurintricarboxylic acid inhibits influenza virus neuraminidase. Antivir. Res..

[51] Barral K., Sallamand C., Petzold C., Coutard B., Collet A., Thillier Y., Zimmermann J., Vasseur J.-J., Canard B., Rohayem J. (2013). Development of specific dengue virus 2′-O- and N7-methyltransferase assays for antiviral drug screening. Antivir. Res..

[52] Falk S.P., Weisblum B. (2014). Aptamer Displacement Screen for Flaviviral RNA Methyltransferase Inhibitors. J. Biomol. Screen..

[53] Chen Y., Bopda-Waffo A., Basu A., Krishnan R., Silberstein E., Taylor D.R., Talele T.T., Arora P., Kaushik-Basu N. (2009). Characterization of aurintricarboxylic acid as a potent hepatitis C virus replicase inhibitor. Antivir. Chem. Chemother..

[54] Mukherjee S., Hanson A.M., Shadrick W.R., Ndjomou J., Sweeney N.L., Hernandez J.J., Bartczak D., Li K., Frankowski K.J., Heck J.A. (2012). Identification and analysis of hepatitis C virus NS3 helicase inhibitors using nucleic acid binding assays. Nucleic Acids Res..

[55] Shadrick W.R., Mukherjee S., Hanson A.M., Sweeney N.L., Frick D.N. (2013). Aurintricarboxylic acid modulates the affinity of hepatitis C virus NS3 helicase for both nucleic acid and ATP. Biochemistry.

[56] Bossart W., Paoletti E., Nuss D.L. (1978). Cell-free translation of purified virion-associated high-molecular-weight RNA synthesized in vitro by vaccinia virus. J. Virol..

[57] Myskiw C., Deschambault Y., Jefferies K., He R., Cao J. (2007). Aurintricarboxylic acid inhibits the early stage of vaccinia virus replication by targeting both cellular and viral factors. J. Virol..

[58] Smee D.F., Hurst B.L., Wong M.-H. (2010). Lack of efficacy of aurintricarboxylic acid and ethacrynic acid against vaccinia virus respiratory infections in mice. Antivir. Chem. Chemother..

[59] Klein P., Cirioni O., Giacometti A., Scalise G. (2008). In vitro and in vivo activity of aurintricarboxylic acid preparations against Cryptosporidium parvum. J. Antimicrob. Chemother..

[60] Liang F., Huang Z., Lee S.-Y., Liang J., Ivanov M.I., Alonso A., Bliska J.B., Lawrence D.S., Mustelin T., Zhang Z.-Y. (2003). Aurintricarboxylic acid blocks in vitro and in vivo activity of YopH, an essential virulent factor of Yersinia pestis, the agent of plague. J. Biol. Chem..

[61] Marín-López A., Bermúdez R., Calvo-Pinilla E., Moreno S., Brun A., Ortego J. (2016). Pathological Characterization Of IFNAR(−/−) Mice Infected With Bluetongue Virus Serotype 4. Int. J. Biol. Sci..

[62] Marín-Lopez A., Calvo-Pinilla E., Moreno S., Utrilla-Trigo S., Nogales A., Brun A., Fikrig E., Ortego J. (2019). Modeling Arboviral Infection in Mice Lacking the Interferon Alpha/Beta Receptor. Viruses.

[63] Mellor P.S., Boorman J., Baylis M. (2000). *Culicoides* Biting Midges: Their Role as Arbovirus Vectors. Annu. Rev. Entomol..

[64] Leta S., Fetene E., Mulatu T., Amenu K., Jaleta M.B., Beyene T.J., Negussie H., Kriticos D., Revie C.W. (2019). Updating the global occurrence of Culicoides imicola, a vector for emerging viral diseases. Sci. Data.

[65] Li Q., Maddox C., Rasmussen L., Hobrath J.V., White L.E. (2009). Assay development and high-throughput antiviral drug screening against Bluetongue virus. Antivir. Res..

[66] Chandra Sekhar K., Venkataramaiah C., Raju C.N. (2020). In silico, in ovo and in vitro antiviral efficacy of phosphorylated derivatives of abacavir: An experimental approach. J. Recept. Signal Transduct. Res..

[67] Darpel K.E., Langner K.F.A., Nimtz M., Anthony S.J., Brownlie J., Takamatsu H.-H., Mellor P.S., Mertens P.P.C. (2011). Saliva proteins of vector Culicoides modify structure and infectivity of bluetongue virus particles. PLoS ONE.

[68] Howerth E.W., Greene C.E., Prestwood A.K. (1988). Experimentally induced bluetongue virus infection in white-tailed deer: Coagulation, clinical pathologic, and gross pathologic changes. Am. J. Vet. Res..

[69] McColl K.A., Gould A.R. (1994). Bluetongue virus infection in sheep: Haematological changes and detection by polymerase chain reaction. Aust. Vet. J..

[70] De la Poza F., Calvo-Pinilla E., López-Gil E., Marín-López A., Mateos F., Castillo-Olivares J., Lorenzo G., Ortego J. (2013). Ns1 is a key protein in the vaccine composition to protect Ifnar(−/−) mice against infection with multiple serotypes of African horse sickness virus. PLoS ONE.

[71] Marín-López A., Calvo-Pinilla E., Barriales D., Lorenzo G., Brun A., Anguita J., Ortego J. (2018). CD8 T Cell Responses to an Immunodominant Epitope within the Nonstructural Protein NS1 Provide Wide Immunoprotection against Bluetongue Virus in IFNAR ^−/−^ Mice. J. Virol..

[72] Calvo-Pinilla E., Rodríguez-Calvo T., Anguita J., Sevilla N., Ortego J. (2009). Establishment of a Bluetongue Virus Infection Model in Mice that Are Deficient in the Alpha/Beta Interferon Receptor. PLoS ONE.

[73] Toussaint J.F., Sailleau C., Breard E., Zientara S., De Clercq K. (2007). Bluetongue virus detection by two real-time RT-qPCRs targeting two different genomic segments. J. Virol. Methods.

[74] Agüero M., Gómez-Tejedor C., Angeles Cubillo M., Rubio C., Romero E., Jiménez-Clavero A. (2008). Real-time fluorogenic reverse transcription polymerase chain reaction assay for detection of African horse sickness virus. J. Vet. Diagn. Investig..

